# Correction: *Histoplasma capsulatum* in wild mammals from Ecuador

**DOI:** 10.1371/journal.pntd.0013708

**Published:** 2025-11-14

**Authors:** Fernanda Hernández-Alomía, Jorge Brito, Ana Lucia Pilatasig, Daniela Reyes-Barriga, Julio C. Carrión-Olmedo, Pablo Jarrín-V, Pablo Sánchez, Santiago F. Burneo, Maria Alejandra Camacho, David Vasco-Julio, Manuel Calvopiña, Jacobus H. De Waard, Daniel Romero-Alvarez, Carlos Bastidas-Caldes

There are errors in the author affiliations. Authors Fernanda Hernández-Alomía and Carlos Bastidas-Caldes were affiliated to Universidad de Las Américas (UDLA) at the time of the study. The UDLA affiliation was omitted in this article [1]. The correct affiliations are as follows:

Fernanda Hernández-Alomía^1¤a^, Jorge Brito^2^, Ana Lucia Pilatasig^3^, Daniela Reyes-Barriga^2^,

Julio C. Carrión-Olmedo^2^, Pablo Jarrín-V^2^, Pablo Sánchez^2^, Santiago F. Burneo^3^,

Maria Alejandra Camacho^3^, David Vasco-Julio^4^, Manuel Calvopiña^5^, Jacobus H. De Waard^5^,

Daniel Romero-Alvarez^2,6^, Carlos Bastidas-Caldes^7,¤b^

**1** Grupo de Investigación en Biodiversidad, Medio Ambiente y Salud (BIOMAS), Universidad de Las Américas, Quito, Ecuador, **2** Instituto Nacional de Biodiversidad (INABIO), Quito, Ecuador, **3** Museo de Zoología, Colección de Mastozoología, Pontificia Universidad Católica del Ecuador, Quito, Ecuador, **4** Laboratorio de Biotecnología Veterinaria, Autopista Manuel Córdoba Galarza, Calacalí, Ecuador, **5** One Health Research Group, Universidad de las Américas, Quito, Ecuador, **6** Research Group of Emerging and Neglected Diseases, Ecoepidemiology and Biodiversity, Health Science Faculty, School of Biomedical Sciences, Universidad Internacional SEK (UISEK), Quito, Ecuador, **7** Faculty of Engineering and Applied Sciences, Biotechnology Engineering Program, Universidad de las Américas, Quito, Ecuador

¤a Current Address: Instituto Nacional de Biodiversidad (INABIO), Quito, Ecuador

¤b Current Address: School of Medicine, Universidad Espíritu Santo, Samborondón, Ecuador
