# Supplementary material for: Histoplasma capsulatum in wild mammals from Ecuador
Source: PLoS Negl Trop Dis. 2025 Aug 25;19(8):e0013410. doi: 10.1371/journal.pntd.0013410 (PMC12404641; doi:10.1371/journal.pntd.0013410)
Supplement: S1 Information — (DOCX) [file pntd.0013410.s001.docx]

**Title:** *Histoplasma capsulatum* in Wild Mammals from Ecuador

**Authors:** Fernanda Hernández-Alomía, Jorge Brito, Ana Pilatasig, Daniela Reyes, Julio C. Carrión-Olmedo, Pablo Jarrín, Pablo Sánchez, Santiago F. Burneo, M. Alejandra Camacho, David Vasco-Julio, Manuel Calvopiña, Jacobus H. De Waard, Daniel Romero-Alvarez, Carlos Bastidas-Caldes

**S1 Information**

**Ecological niche models**

**Occurrences**

We used positive detections of *H. capsulatum* as working occurrences for the ecological niche modeling (1). As our sampling is far from comprehensive considering species collected, geographic breadth, and collection of tissues through time, absence data is fully speculative and halts interpretability beyond the identification of clusters of environmental similarity (2). Consequently, we used a presence-only approach using hypervolumes (3).

**Environmental predictors**

As spore survival depends on particular environmental contexts (4), we used temperature, relative humidity, precipitation, and chemical soil characteristics to suggest potential areas of *H. capsulatum* suitability in Ecuador. For temperature and relative humidity, we used bioclimatic variables derived from the MERRAclim database (5) which summarizes temperature and relative humidity values for 2000-2010 derived from satellite data at 5 arc-min pixel resolution. We avoided raster files combining temperature and humidity as they have artifacts (6). Precipitation was obtained as a raster layer from the CHELSA repository at 5 arc-minute resolution as mm/month averaging information from 1979-2013 (7). For soil, we included variables describing chemical characteristics at 0-5 cm depth at 250 m pixel resolution; specifically, we used cation exchange capacity, organic soil carbon, water pH, and nitrogen obtained from the SoilGrids repository (8). Temperature (n=9), relative humidity (n=6), soil (n=4), and precipitation (n=1) accounted for a total of 20 environmental predictors for modeling. When needed, predictors were resampled to 5 arc-minute resolution. We used a single value decomposition principal component analysis (PCA) to control for multicollinearity which is known for overfitting modeling outputs (9). The principal components (PCs) with >85% of the variance were used as final predictors. Manipulation of raster files and PCA were done with packages ‘raster’, ‘terra’, and ‘kunem’ in R programming language, version 4.3.2 (10).

**Algorithm**

One class support vector machine (OC-SVM) hypervolumes were developed as an algorithm for detecting clusters around subsets of available occurrences but in the environmental space (3). OC-SVM builds an hyper ellipse around support vectors selected from the occurrences, this hyper ellipse is later trimmed based on default parameters to suggest the most optimal environmental clusters that encompass the data available (3). We used the binary output of the algorithm for interpretation of the geographic projection in Ecuador.

**Final model**

We developed models using n-1 points until all the available occurrences were used for model building. We built 11 models based on *H. capsulatum* detections in Ecuador. Outputs have a binary value of presence (=1) and absence (=0) suggesting whether environmental conditions were suitable or unsuitable for the fungi. We added each model to obtain a gridded output with values ranging from zero to eleven showing the number of times a pixel was identified as suitable by any of the models. We categorized the summed output in low, moderate, and high suitability considering pixel agreement between 1 to 3 models, 4 to 6, or 7 to 11, respectively.

**Supplementary references**

1. Peterson AT. Mapping Disease Transmission Risk: Enriching Models Using Biogeography and Ecology. Baltimore: Johns Hopkins University Press; 2014.

2. Peterson AT, Soberón J, Pearson RG, Anderson RP, Martínez-Meyer E, Nakamura M, et al. Ecological Niches and Geographic Distributions. New Jersey: Princeton University Press; 2011.

3. Blonder B, Morrow CB, Maitner B, Harris DJ, Lamanna C, Violle C, et al. New approaches for delineating n-dimensional hypervolumes. Methods in Ecology and Evolution. 2018;9:305–19.

4. Taylor ML, Reyes-Montes MDR, Estrada-Bárcenas DA, Zancopé-Oliveira RM, Rodríguez-Arellanes G, Ramírez JA. Considerations about the geographic distribution of *Histoplasma* species. Applied and Environmental Microbiology. 2022;88:e02010-21.

5. Vega GC, Pertierra LR, Olalla-Táraga MÁ. MERRAclim, a high-resolution global dataset of remotely sensed bioclimatic variables for ecological modelling. Scientific Data. 2017;4:170078.

6. Booth TH. Checking bioclimatic variables that combine temperature and precipitation data before their use in species distribution models. Austral Ecology. 2022;47:1506–14.

7. Karger DN, Conrad O, Böhner J, Kawohl T, Kreft H, Soria-Auza RW, et al. Climatologies at high resolution for the earth’s land surface areas. Scientific Data. 2017;4:170122.

8. Hengl T, Mendes de Jesus J, Heuvelink GBM, Ruiperez Gonzalez M, Kilibarda M, Blagotić A, et al. SoilGrids250m: global gridded soil information based on machine learning. PLoS one. 2017;12:e0169748.

9. Cobos ME, Peterson AT, Osorio-Olvera L, Jiménez-García D. An exhaustive analysis of heuristic methods for variable selection in ecological niche modeling and species distribution modeling. Ecological Informatics. 2019;53:100983.

10. R Core Team. R: A language and environment for statistical computing. Vienna, Austria: R Foundation for Statistical Computing; 2023. Available: https://www.r-project.org/
