# Supplementary material for: Histoplasma capsulatum in wild mammals from Ecuador
Source: PLoS Negl Trop Dis. 2025 Aug 25;19(8):e0013410. doi: 10.1371/journal.pntd.0013410 (PMC12404641; doi:10.1371/journal.pntd.0013410)
Supplement: Attachment — Submitted filename: hc_100.nwk2.txt.pdf [file pntd.0013410.s005.pdf]

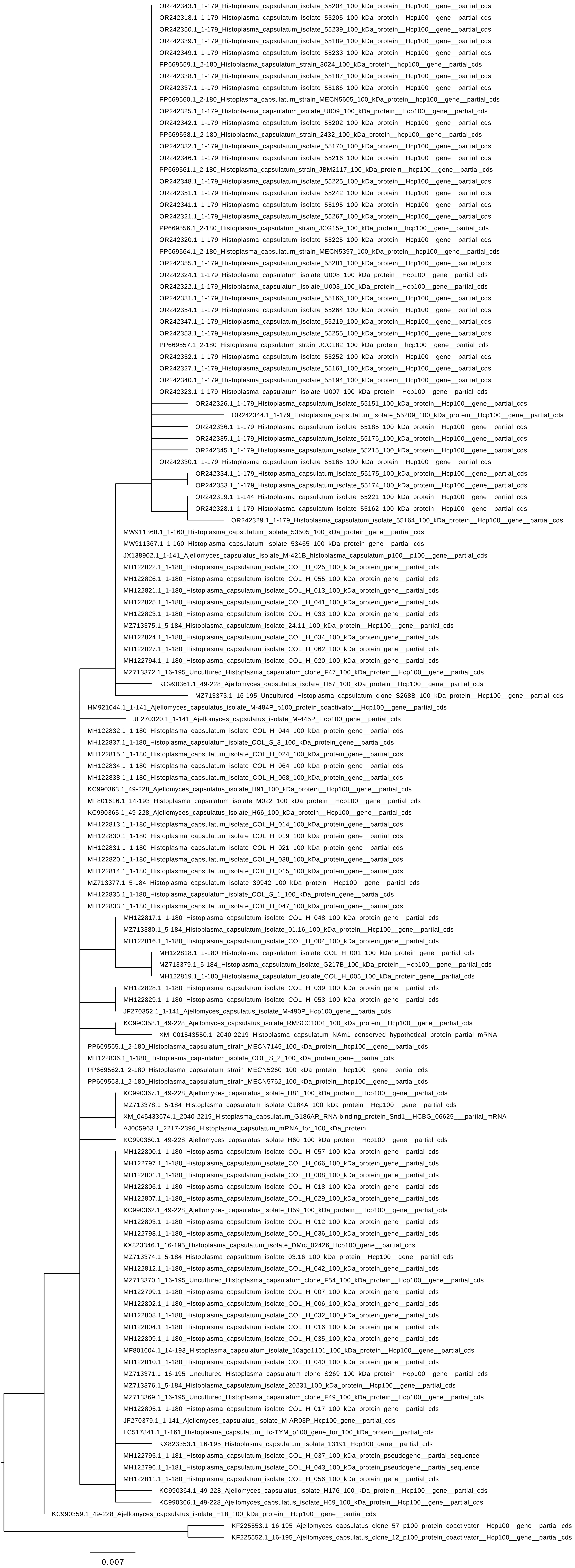
OR242343.1_1-179_Histoplasma_capsulatum_isolate_55204_100_kDa_protein__Hcp100__gene__partial_cds
OR242318.1_1-179_Histoplasma_capsulatum_isolate_55205_100_kDa_protein__Hcp100__gene__partial_cds
OR242350.1_1-179_Histoplasma_capsulatum_isolate_55239_100_kDa_protein__Hcp100__gene__partial_cds
OR242339.1_1-179_Histoplasma_capsulatum_isolate_55189_100_kDa_protein__Hcp100__gene__partial_cds
OR242349.1_1-179_Histoplasma_capsulatum_isolate_55233_100_kDa_protein__Hcp100__gene__partial_cds
PP669559.1_2-180_Histoplasma_capsulatum_strain_3024_100_kDa_protein__hcp100__gene__partial_cds
OR242338.1_1-179_Histoplasma_capsulatum_isolate_55187_100_kDa_protein__Hcp100__gene__partial_cds
OR242337.1_1-179_Histoplasma_capsulatum_isolate_55186_100_kDa_protein__Hcp100__gene__partial_cds
PP669560.1_2-180_Histoplasma_capsulatum_strain_MECN5605_100_kDa_protein__hcp100__gene__partial_cds
OR242325.1_1-179_Histoplasma_capsulatum_isolate_U009_100_kDa_protein__Hcp100__gene__partial_cds
OR242342.1_1-179_Histoplasma_capsulatum_isolate_55202_100_kDa_protein__Hcp100__gene__partial_cds
PP669558.1_2-180_Histoplasma_capsulatum_strain_2432_100_kDa_protein__hcp100__gene__partial_cds
OR242332.1_1-179_Histoplasma_capsulatum_isolate_55170_100_kDa_protein__Hcp100__gene__partial_cds
OR242346.1_1-179_Histoplasma_capsulatum_isolate_55216_100_kDa_protein__Hcp100__gene__partial_cds
PP669561.1_2-180_Histoplasma_capsulatum_strain_JBM2117_100_kDa_protein__hcp100__gene__partial_cds
OR242348.1_1-179_Histoplasma_capsulatum_isolate_55225_100_kDa_protein__Hcp100__gene__partial_cds
OR242351.1_1-179_Histoplasma_capsulatum_isolate_55242_100_kDa_protein__Hcp100__gene__partial_cds
OR242341.1_1-179_Histoplasma_capsulatum_isolate_55195_100_kDa_protein__Hcp100__gene__partial_cds
OR242321.1_1-179_Histoplasma_capsulatum_isolate_55267_100_kDa_protein__Hcp100__gene__partial_cds
PP669556.1_2-180_Histoplasma_capsulatum_strain_JCG159_100_kDa_protein__hcp100__gene__partial_cds
OR242320.1_1-179_Histoplasma_capsulatum_isolate_55225_100_kDa_protein__Hcp100__gene__partial_cds
PP669564.1_2-180_Histoplasma_capsulatum_strain_MECN5397_100_kDa_protein__hcp100__gene__partial_cds
OR242355.1_1-179_Histoplasma_capsulatum_isolate_55281_100_kDa_protein__Hcp100__gene__partial_cds
OR242324.1_1-179_Histoplasma_capsulatum_isolate_U008_100_kDa_protein__Hcp100__gene__partial_cds
OR242322.1_1-179_Histoplasma_capsulatum_isolate_U003_100_kDa_protein__Hcp100__gene__partial_cds
OR242331.1_1-179_Histoplasma_capsulatum_isolate_55166_100_kDa_protein__Hcp100__gene__partial_cds
OR242354.1_1-179_Histoplasma_capsulatum_isolate_55264_100_kDa_protein__Hcp100__gene__partial_cds
OR242347.1_1-179_Histoplasma_capsulatum_isolate_55219_100_kDa_protein__Hcp100__gene__partial_cds
OR242353.1_1-179_Histoplasma_capsulatum_isolate_55255_100_kDa_protein__Hcp100__gene__partial_cds
PP669557.1_2-180_Histoplasma_capsulatum_strain_JCG182_100_kDa_protein__hcp100__gene__partial_cds
OR242352.1_1-179_Histoplasma_capsulatum_isolate_55252_100_kDa_protein__Hcp100__gene__partial_cds
OR242327.1_1-179_Histoplasma_capsulatum_isolate_55161_100_kDa_protein__Hcp100__gene__partial_cds
OR242340.1_1-179_Histoplasma_capsulatum_isolate_55194_100_kDa_protein__Hcp100__gene__partial_cds
OR242323.1_1-179_Histoplasma_capsulatum_isolate_U007_100_kDa_protein__Hcp100__gene__partial_cds
OR242326.1_1-179_Histoplasma_capsulatum_isolate_55151_100_kDa_protein__Hcp100__gene__partial_cds
OR242344.1_1-179_Histoplasma_capsulatum_isolate_55209_100_kDa_protein__Hcp100__gene__partial_cds
OR242336.1_1-179_Histoplasma_capsulatum_isolate_55185_100_kDa_protein__Hcp100__gene__partial_cds
OR242335.1_1-179_Histoplasma_capsulatum_isolate_55176_100_kDa_protein__Hcp100__gene__partial_cds
OR242345.1_1-179_Histoplasma_capsulatum_isolate_55215_100_kDa_protein__Hcp100__gene__partial_cds
OR242330.1_1-179_Histoplasma_capsulatum_isolate_55165_100_kDa_protein__Hcp100__gene__partial_cds
OR242334.1_1-179_Histoplasma_capsulatum_isolate_55175_100_kDa_protein__Hcp100__gene__partial_cds
OR242333.1_1-179_Histoplasma_capsulatum_isolate_55174_100_kDa_protein__Hcp100__gene__partial_cds
OR242319.1_1-144_Histoplasma_capsulatum_isolate_55221_100_kDa_protein__Hcp100__gene__partial_cds
OR242328.1_1-179_Histoplasma_capsulatum_isolate_55162_100_kDa_protein__Hcp100__gene__partial_cds
OR242329.1_1-179_Histoplasma_capsulatum_isolate_55164_100_kDa_protein__Hcp100__gene__partial_cds
MW911368.1_1-160_Histoplasma_capsulatum_isolate_53505_100_kDa_protein_gene__partial_cds
MW911367.1_1-160_Histoplasma_capsulatum_isolate_53465_100_kDa_protein_gene__partial_cds
JX138902.1_1-141_Ajellomyces_capsulatus_isolate_M-421B_histoplasma_capsulatum_p100__p100__gene__partial_cds
MH122822.1_1-180_Histoplasma_capsulatum_isolate_COL_H_025_100_kDa_protein_gene__partial_cds
MH122826.1_1-180_Histoplasma_capsulatum_isolate_COL_H_055_100_kDa_protein_gene__partial_cds
MH122821.1_1-180_Histoplasma_capsulatum_isolate_COL_H_013_100_kDa_protein_gene__partial_cds
MH122825.1_1-180_Histoplasma_capsulatum_isolate_COL_H_041_100_kDa_protein_gene__partial_cds
MH122823.1_1-180_Histoplasma_capsulatum_isolate_COL_H_033_100_kDa_protein_gene__partial_cds
MZ713375.1_5-184_Histoplasma_capsulatum_isolate_24.11_100_kDa_protein__Hcp100__gene__partial_cds
MH122824.1_1-180_Histoplasma_capsulatum_isolate_COL_H_034_100_kDa_protein_gene__partial_cds
MH122827.1_1-180_Histoplasma_capsulatum_isolate_COL_H_062_100_kDa_protein_gene__partial_cds
MH122794.1_1-180_Histoplasma_capsulatum_isolate_COL_H_020_100_kDa_protein_gene__partial_cds
MZ713372.1_16-195_Uncultured_Histoplasma_capsulatum_clone_F47_100_kDa_protein__Hcp100__gene__partial_cds
KC990361.1_49-228_Ajellomyces_capsulatus_isolate_H67_100_kDa_protein__Hcp100__gene__partial_cds
MZ713373.1_16-195_Uncultured_Histoplasma_capsulatum_clone_S268B_100_kDa_protein__Hcp100__gene__partial_cds
HM921044.1_1-141_Ajellomyces_capsulatus_isolate_M-484P_p100_protein_coactivator__Hcp100__gene__partial_cds
JF270320.1_1-141_Ajellomyces_capsulatus_isolate_M-445P_Hcp100_gene__partial_cds
MH122832.1_1-180_Histoplasma_capsulatum_isolate_COL_H_044_100_kDa_protein_gene__partial_cds
MH122837.1_1-180_Histoplasma_capsulatum_isolate_COL_S_3_100_kDa_protein_gene__partial_cds
MH122815.1_1-180_Histoplasma_capsulatum_isolate_COL_H_024_100_kDa_protein_gene__partial_cds
MH122834.1_1-180_Histoplasma_capsulatum_isolate_COL_H_064_100_kDa_protein_gene__partial_cds
MH122838.1_1-180_Histoplasma_capsulatum_isolate_COL_H_068_100_kDa_protein_gene__partial_cds
KC990363.1_49-228_Ajellomyces_capsulatus_isolate_H91_100_kDa_protein__Hcp100__gene__partial_cds
MF801616.1_14-193_Histoplasma_capsulatum_isolate_M022_100_kDa_protein__Hcp100__gene__partial_cds
KC990365.1_49-228_Ajellomyces_capsulatus_isolate_H66_100_kDa_protein__Hcp100__gene__partial_cds
MH122813.1_1-180_Histoplasma_capsulatum_isolate_COL_H_014_100_kDa_protein_gene__partial_cds
MH122830.1_1-180_Histoplasma_capsulatum_isolate_COL_H_019_100_kDa_protein_gene__partial_cds
MH122831.1_1-180_Histoplasma_capsulatum_isolate_COL_H_021_100_kDa_protein_gene__partial_cds
MH122820.1_1-180_Histoplasma_capsulatum_isolate_COL_H_038_100_kDa_protein_gene__partial_cds
MH122814.1_1-180_Histoplasma_capsulatum_isolate_COL_H_015_100_kDa_protein_gene__partial_cds
MZ713377.1_5-184_Histoplasma_capsulatum_isolate_39942_100_kDa_protein__Hcp100__gene__partial_cds
MH122835.1_1-180_Histoplasma_capsulatum_isolate_COL_S_1_100_kDa_protein_gene__partial_cds
MH122833.1_1-180_Histoplasma_capsulatum_isolate_COL_H_047_100_kDa_protein_gene__partial_cds
MH122817.1_1-180_Histoplasma_capsulatum_isolate_COL_H_048_100_kDa_protein_gene__partial_cds
MZ713380.1_5-184_Histoplasma_capsulatum_isolate_01.16_100_kDa_protein__Hcp100__gene__partial_cds
MH122816.1_1-180_Histoplasma_capsulatum_isolate_COL_H_004_100_kDa_protein_gene__partial_cds
MH122818.1_1-180_Histoplasma_capsulatum_isolate_COL_H_001_100_kDa_protein_gene__partial_cds
MZ713379.1_5-184_Histoplasma_capsulatum_isolate_G217B_100_kDa_protein__Hcp100__gene__partial_cds
MH122819.1_1-180_Histoplasma_capsulatum_isolate_COL_H_005_100_kDa_protein_gene__partial_cds
MH122828.1_1-180_Histoplasma_capsulatum_isolate_COL_H_039_100_kDa_protein_gene__partial_cds
MH122829.1_1-180_Histoplasma_capsulatum_isolate_COL_H_053_100_kDa_protein_gene__partial_cds
JF270352.1_1-141_Ajellomyces_capsulatus_isolate_M-490P_Hcp100_gene__partial_cds
KC990358.1_49-228_Ajellomyces_capsulatus_isolate_RMSCC1001_100_kDa_protein__Hcp100__gene__partial_cds
XM_001543550.1_2040-2219_Histoplasma_capsulatum_NAm1_conserved_hypothetical_protein_partial_mRNA
PP669565.1_2-180_Histoplasma_capsulatum_strain_MECN7145_100_kDa_protein__hcp100__gene__partial_cds
MH122836.1_1-180_Histoplasma_capsulatum_isolate_COL_S_2_100_kDa_protein_gene__partial_cds
PP669562.1_2-180_Histoplasma_capsulatum_strain_MECN5260_100_kDa_protein__hcp100__gene__partial_cds
PP669563.1_2-180_Histoplasma_capsulatum_strain_MECN5762_100_kDa_protein__hcp100__gene__partial_cds
KC990367.1_49-228_Ajellomyces_capsulatus_isolate_H81_100_kDa_protein__Hcp100__gene__partial_cds
MZ713378.1_5-184_Histoplasma_capsulatum_isolate_G184A_100_kDa_protein__Hcp100__gene__partial_cds
XM_045433674.1_2040-2219_Histoplasma_capsulatum_G186AR_RNA-binding_protein_Snd1__HCBG_06625___partial_mRNA
AJ005963.1_2217-2396_Histoplasma_capsulatum_mRNA_for_100_kDa_protein
KC990360.1_49-228_Ajellomyces_capsulatus_isolate_H60_100_kDa_protein__Hcp100__gene__partial_cds
MH122800.1_1-180_Histoplasma_capsulatum_isolate_COL_H_057_100_kDa_protein_gene__partial_cds
MH122797.1_1-180_Histoplasma_capsulatum_isolate_COL_H_066_100_kDa_protein_gene__partial_cds
MH122801.1_1-180_Histoplasma_capsulatum_isolate_COL_H_008_100_kDa_protein_gene__partial_cds
MH122806.1_1-180_Histoplasma_capsulatum_isolate_COL_H_018_100_kDa_protein_gene__partial_cds
MH122807.1_1-180_Histoplasma_capsulatum_isolate_COL_H_029_100_kDa_protein_gene__partial_cds
KC990362.1_49-228_Ajellomyces_capsulatus_isolate_H59_100_kDa_protein__Hcp100__gene__partial_cds
MH122803.1_1-180_Histoplasma_capsulatum_isolate_COL_H_012_100_kDa_protein_gene__partial_cds
MH122798.1_1-180_Histoplasma_capsulatum_isolate_COL_H_036_100_kDa_protein_gene__partial_cds
KX823346.1_16-195_Histoplasma_capsulatum_isolate_DMic_02426_Hcp100_gene__partial_cds
MZ713374.1_5-184_Histoplasma_capsulatum_isolate_03.16_100_kDa_protein__Hcp100__gene__partial_cds
MH122812.1_1-180_Histoplasma_capsulatum_isolate_COL_H_042_100_kDa_protein_gene__partial_cds
MZ713370.1_16-195_Uncultured_Histoplasma_capsulatum_clone_F54_100_kDa_protein__Hcp100__gene__partial_cds
MH122799.1_1-180_Histoplasma_capsulatum_isolate_COL_H_007_100_kDa_protein_gene__partial_cds
MH122802.1_1-180_Histoplasma_capsulatum_isolate_COL_H_006_100_kDa_protein_gene__partial_cds
MH122808.1_1-180_Histoplasma_capsulatum_isolate_COL_H_032_100_kDa_protein_gene__partial_cds
MH122804.1_1-180_Histoplasma_capsulatum_isolate_COL_H_016_100_kDa_protein_gene__partial_cds
MH122809.1_1-180_Histoplasma_capsulatum_isolate_COL_H_035_100_kDa_protein_gene__partial_cds
MF801604.1_14-193_Histoplasma_capsulatum_isolate_10ago1101_100_kDa_protein__Hcp100__gene__partial_cds
MH122810.1_1-180_Histoplasma_capsulatum_isolate_COL_H_040_100_kDa_protein_gene__partial_cds
MZ713371.1_16-195_Uncultured_Histoplasma_capsulatum_clone_S269_100_kDa_protein__Hcp100__gene__partial_cds
MZ713376.1_5-184_Histoplasma_capsulatum_isolate_20231_100_kDa_protein__Hcp100__gene__partial_cds
MZ713369.1_16-195_Uncultured_Histoplasma_capsulatum_clone_F49_100_kDa_protein__Hcp100__gene__partial_cds
MH122805.1_1-180_Histoplasma_capsulatum_isolate_COL_H_017_100_kDa_protein_gene__partial_cds
JF270379.1_1-141_Ajellomyces_capsulatus_isolate_M-AR03P_Hcp100_gene__partial_cds
LC517841.1_1-161_Histoplasma_capsulatum_Hc-TYM_p100_gene_for_100_kDa_protein__partial_cds
KX823353.1_16-195_Histoplasma_capsulatum_isolate_13191_Hcp100_gene__partial_cds
MH122795.1_1-181_Histoplasma_capsulatum_isolate_COL_H_037_100_kDa_protein_pseudogene__partial_sequence
MH122796.1_1-181_Histoplasma_capsulatum_isolate_COL_H_043_100_kDa_protein_pseudogene__partial_sequence
MH122811.1_1-180_Histoplasma_capsulatum_isolate_COL_H_056_100_kDa_protein_gene__partial_cds
KC990364.1_49-228_Ajellomyces_capsulatus_isolate_H176_100_kDa_protein__Hcp100__gene__partial_cds
KC990366.1_49-228_Ajellomyces_capsulatus_isolate_H69_100_kDa_protein__Hcp100__gene__partial_cds
KC990359.1_49-228_Ajellomyces_capsulatus_isolate_H18_100_kDa_protein__Hcp100__gene__partial_cds
KF225553.1_16-195_Ajellomyces_capsulatus_clone_57_p100_protein_coactivator__Hcp100__gene__partial_cds
KF225552.1_16-195_Ajellomyces_capsulatus_clone_12_p100_protein_coactivator__Hcp100__gene__partial_cds
0.007
