# Supplementary material for: Histoplasma capsulatum in wild mammals from Ecuador
Source: PLoS Negl Trop Dis. 2025 Aug 25;19(8):e0013410. doi: 10.1371/journal.pntd.0013410 (PMC12404641; doi:10.1371/journal.pntd.0013410)
Supplement: Attachment — Submitted filename: Response Letter_PlosNTD.docx [file pntd.0013410.s008.docx]

**Response Letter**

**PNTD-D-25-00302**

**Histoplasma capsulatum in Wild Mammals from Ecuador**

PLOS Neglected Tropical Diseases

Dear Editor,

We have carefully reviewed the manuscript according to the comments and suggestions provided by Reviewers 1, 2, and 3. Our responses are included immediately after each observation and highlighted in blue for clarity. We sincerely thank the reviewers and the editor for their valuable feedback and appreciate the opportunity to improve our work. We hope the revised manuscript meets their expectations. All changes in the manuscript are clearly marked for ease of review.

**Methods**

**Reviewer #1:** Phylogenetic analysis are not

**Response**: We have revised the manuscript to enhance the molecular identification of *H. capsulatum*. All comments and observations made by Reviewer 1 have been thoroughly addressed in the response letter below. We sincerely thank you for your valuable feedback.

**Reviewer #2:** Methods are not clear. Justification for collecting tissues (result.s site lung, liver, spleen and feces) but presenting on only liver in Table 1 is confusing. were other tissues not screened? Screened but not positive?

**Response**: We thank the reviewer for this insightful observation and fully understand their concern. To avoid any confusion, we have revised and clarified the wording of the corresponding section. In the “Methodology, Sample and Site Specimen Collection” section, we now specify eco regions of Ecuador where specimen sampling was conducted.

Tissue collection leverages the ongoing work of natural science and zoology museums to conduct wildlife surveillance for *H. capsulatum*, in alignment with *Primary Pandemic Prevention* efforts (REF 22). This rationale has been explicitly incorporated at the end of the Introduction section to frame the public health relevance of the study. It is important to note that euthanized animals are preserved as part of the permanent collections of the Ecuadorian National Biodiversity Institute (INABIO, in Spanish) and the Museum of Zoology of the Pontifical Catholic University of Ecuador (QCAZ, in Spanish), which routinely process and collect samples from wildlife as part of their monitoring activities. Our research is maximizing the utility of these samples via infectious disease surveillance.

For armadillos, tissues were obtained as donations from hunters in communities that consume their meat. Although the Ecuadorian Constitution and laws prohibit the consumption of wild meat, this practice remains common in Ecuador, is poorly controlled, and in some rural areas it persists as an accessible source of protein. Regarding Table 1 in the Results section, we have clarified that it includes only those tissues that tested positive for *H. capsulatum*. Although molecular testing was performed on all described tissues and samples, only liver and fecal samples yielded positive results. Furthermore, all animals and tissue samples—regardless of their *H. capsulatum* status—are listed in Supplementary Table 1, which is cited at the end of the first paragraph of the Results section.

REF 22: Plowright RK, Ahmed AN, Coulson T, Crowther TW, Ejotre I, Faust CL, et al. Ecological countermeasures to prevent pathogen spillover and subsequent pandemics. Nature Communications 2024 15:1. 2024;15: 1–13. doi:10.1038/s41467-024-46151-9

There is biased sampling, quantity of animals captured in one region may bias results.

**Response**: We strongly agree with the reviewer. The capture and availability of wild animals is affected by sampling effort, climatic season, species status, and other factors that are complicated to manage in the field. However, our intention is to demonstrate the wide distribution of *H. capsulatum* in wild mammals. In this context, we decided to perform the analyses on all available animal samples from the participating institutions. In the “Study limitations” section, we added this important detail.

Global Chi-square test may be inappropriate given the uneven distribution of species. what is the true abundance of the species in question? This test is assuming that you captured a representative set of host orders. wouldn't you test each order separately?

**Response**: We agree with the reviewer. For this version we have improved the statistical analysis. We are aware of the existing bias in the representativeness of individuals for each order; however, other factors related to sampling such as conservation status of the species, habitat status, accessibility to collection areas should be considered as further sources of biases. Consequently, for this version we have improved the analysis as follows: we applied an overall Chi-square and individual Chi-squares of positive *H. capsulatum* orders to determine whether the prevalence of *H. capsulatum* varied significantly among wild mammal taxonomic orders. The null hypothesis assumed no association between host order and fungal detection status. Statistical significance was defined as *p* < 0.05.

Approval for trapping and euthanasia was stated in manuscript

**Response**: Yes. We have collection permits from the Ecuadorian National Institute of Biodiversity (INABIO, in Spanish). We also have collection permits of the Museum of Zoology of the Pontifical Catholic University of Ecuador (QCAZ, in Spanish). The euthanasia protocols are those established by each museum following the rules established in the regulations of the Ministry of Environment, Water and Ecological Transition of Ecuador (MAATE).

**Reviewer #3**:

-Are the objectives of the study clearly articulated with a clear testable hypothesis stated? Yes

-Is the study design appropriate to address the stated objectives?

Comment: I would like to question the efficacy of the Hcp100 molecular marker for the phylogenetic analysis. The Hcp100 molecular marker is successfully used in clinical diagnosis and for searching the H. capsulatum presence in randomly infected animals, because this marker amplified a specific DNA fragment of 210 bp, in the second Nested-PCR reaction, which is unique for this fungal pathogen. Although its use for H. capsulatum phylogenetic analyses is not the most recommendable, mainly considering that the Hcp100 fragment presents very low polymorphism, clades obtained were certainly representative and discriminate the different groups of animals studied. Thus, I suggest including an appropriate interpretation in the Discussion section of the Manuscript.

**Response**: We appreciate the reviewer’s valuable comment. We agree that the efficacy of the Hcp100 marker lies in the molecular identification of *H. capsulatum* and acknowledge its limited phylogenetic signal. Following your recommendation we have decided to remove phylogenetic analyses in the study. The sequences obtained from the second-round nested PCR products of Hcp100 are retained as evidence of the molecular identification of *H. capsulatum* (Supplementary Table 3). In the Results section, the description of the molecular identification has now been revised and is presented solely as the molecular identification of the pathogen in wild mammals.

-Is the population clearly described and appropriate for the hypothesis being tested? Yes
-Is the sample size sufficient to ensure adequate power to address the hypothesis being tested? Yes
-Were correct statistical analysis used to support conclusions? Please, see my comment: In regarding to the revision of the Manuscript MS PNTD-D-25-0030, entitled
"Histoplasma capsulatum in Wild Mammals from Ecuador", I would like to
emphasize its importance in the epidemiology of histoplasmosis, mainly
considering a wide sort of animals studied (106 species) and the role of wild
mammals as possible reservoirs and, particularly, the role of bats as the main
reservoir and disperser of the fungal pathogen H. capsulatum in nature, as have
been suggested by some researchers. Thus, any type of evidence for the
occurrence of H. capsulatum in wild mammals, either in Latin America or in other
regions or the world should be privileged. However, I would like to question the
efficacy of the Hcp100 molecular marker for the phylogenetic analysis. The Hcp100
molecular marker is successfully used in clinical diagnosis and for searching the H.
capsulatum presence in randomly infected animals, because this marker amplified
a specific DNA fragment of 210 bp, in the second Nested-PCR reaction, which is
unique for this fungal pathogen. Although its use for H. capsulatum phylogenetic
analyses is not the most recommendable, mainly considering that the Hcp100
fragment presents very low polymorphism, clades obtained were certainly
representative and discriminate the different groups of animals studied. Hence, I
suggest including appropriate interpretations in the Discussion section of the
Manuscript.
Overall, considering the above-comment, I think that the Manuscript must be
accepted.

**Response**: We greatly appreciate and value your feedback. We refer the reviewer to the above comment for an appropriate response.

-Are there concerns about ethical or regulatory requirements being met? Yes

**Results**

**Reviewer #1:** Phylogenetic analysis are not

**Response**: Please see the improved results section in the manuscript.

**Reviewer #2:** Percentage positivity is slightly misleading when only one representative of the species was captured.

**Response**: Thank you very much for the comment, we agree, statistically there may be a bias. However, the study shows the prevalence of a great diversity of mammals (n=106 species), it is very difficult to have a representative sample of each of them. In addition, the actual sampling is limited by other factors such as conservation status of the species (juvenile, pregnant or at risk of extinction specimens), habitat status, and accessibility to collection areas. Despite this limitation, we believe that our findings provide important information on the status and spread of *H. capsulatum* and its reservoirs precisely because our analysis encompasses 106 mammal species.

Data in supplemental table doesn't seem to match Table 1 - what is the status of samples 294 and 317? is this collected from animal directly or was this from the cave floor? If the latter, this is an environmental sample}

**Response**: We are very grateful for the comment. We have modified Table 1 and now include samples 261, 294, and 317, whose species were unknown. These samples are now categorized as “unidentified” at the end of table 1. The stool samples were obtained directly from the animal.

Due to the high similarity of these sequences and having only a single sequence from PCR, a phylogenetic tree is likely a poor methods to describe diversity. this is a ~180 bp fragment, so percent identity would be clearer. no branch support reported and provides little information on Histoplasma diversity in Ecuador.

**Response**: We appreciate the reviewer’s valuable comment. We agree that the efficacy of the Hcp100 marker lies in the molecular identification of *H. capsulatum* and acknowledge its limited phylogenetic signal. Thus, for this version we have eliminated the phylogenetic analysis. The sequences obtained from the second-round nested PCR products of Hcp100 are retained as evidence of the molecular identification of *H. capsulatum* (Supplementary Table 3). In the Results section, the description of the molecular identification has now been revised and is presented solely as the molecular identification of the pathogen in wild mammals.

The ecological niche model is not well described and could simply model species distribution rather that fungal distribution. Very confusing.

Response: Thanks for bringing this topic to the review. For the ecological niche modelling we are using a presence-only algorithm to estimate the potential distribution of the species studied, in this case *H. capsulatum*. We are treating the pathogen presence across reservoirs as the unit of analysis and therefore suggesting the potential environmental conditions that, in this study, represent suitable conditions for pathogen-spore survival. As our data is constrained to sampling availability collected for different purposes, there is a bias in our approach highlighted in the discussion and limitation sections. However, as systematic collection of mammals is seldom performed in Ecuador plus the scarce information of *H. capsulatum* in wildlife, we believe our modelling approach offers an opportunity to highlight regions for future mammal surveillance for this infectious disease system.

Regarding the model, we believe it is clearly described. Methods are mentioning information relevant to replicate the experiment, including: the algorithm used, its applicability, the assumption behind the model (i.e., *H. capsulatum* spore survival), the environmental variables selected and how they were processed (i.e., PCA). Moreover, in the supplementary material we expand the discussion of each of these parameters, and we clearly justify each modelling step with ten selected references that are available independently in the supplementary material. Moreover, the results section is describing overall patterns of suitability as it is also the standard in the field. Finally, the discussion section is mentioning both the limitations and opportunities offered by this kind of models for understanding the potential distribution of pathogens for future surveillance studies. Thanks!

**Reviewer #3:** -Does the analysis presented match the analysis plan? Please, see my comment

-Are the results clearly and completely presented? Delete repetitive phrases in the Results section, I.e.: lines 240-242, - Sequences marked with an asterisk (PP669556, PP669558, OR242338) represent identical sequences found in other specimens analyzed in this study. Sequences marked with an asterisk (PP669556, 242 PP669558, OR242338) represent identical sequences found in other specimens analyzed in this study

**Response**: Thank you very much. Done.

-Are the figures (Tables, Images) of sufficient quality for clarity? Yes

**Conclusions**

**Reviewer #1:** yes

**Reviewer #2:** The discussion is diffuse and sometimes confusing. The point that we know little about the range of potential infected animals is key, and I think the data collected support that. Focus on this. I think some statement of the limitations of the study and future directions would be useful.

**Response**: Thank you very much for your comment. We have revised and improved the entire discussion section and included a section on study limitations to comply with the reviewer’s request.

**Reviewer #3:** -Are the conclusions supported by the data presented? Yes
-Are the limitations of analysis clearly described? Please, see my comment
-Do the authors discuss how these data can be helpful to advance our understanding of the topic under study? Please, see my comment
-Is public health relevance addressed? Yes

**Response**: Thank you very much for the comment and we appreciate your feedback. Please refer to the comment above. We have discussed the Hcp100 molecular in previous responses. More importantly, for this version we have improved the results and discussion section.

**Editorial and Data Presentation Modifications?**

**Reviewer #1:** (No Response)

**Reviewer #2:** There must be clearer description of methods, justification of tissues samples and improved statistical analysis, as suggested above.

**Response**: Done

**Reviewer #3:** Minor observation:

Delete repetitive phrases I.e.: pages 240-242, - Sequences marked with an asterisk (PP669556, PP669558, OR242338) represent identical sequences found in other specimens analyzed in
this study. Sequences marked with an asterisk (PP669556, 242 PP669558,
OR242338) represent identical sequences found in other specimens analyzed in
this study

**Response**: Done

**Summary and General Comments**

Use this section to provide overall comments, discuss strengths/weaknesses of the study, novelty, significance, general execution and scholarship. You may also include additional comments for the author, including concerns about dual publication, research ethics, or publication ethics. If requesting major revision, please articulate the new experiments that are needed.

**Reviewer #1:** This manuscript addresses a highly relevant and timely topic by investigating Histoplasma capsulatum infection in a variety of wild mammalian hosts across Ecuador. The study provides valuable insights into the ecological distribution and potential zoonotic interfaces of H. capsulatum, contributing to ongoing discussions in medical mycology and One Health frameworks. However, while the core objectives are important, the manuscript still requires significant revisions to improve scientific rigor, methodological transparency, and interpretative depth. Several sections—including the abstract, introduction, methods, phylogenetic analysis, and discussion—would benefit from restructuring, clarification, and more robust contextualization within the existing literature. I outline specific comments and suggestions below to help the authors enhance the overall clarity, accuracy, and impact of their work.

Abstract:
Line 28 – The statement “is disseminated by avian and chiropteran reservoirs” appears to be overly speculative, particularly regarding avian hosts. While Histoplasma has been consistently detected in association with chiropteran reservoirs—supporting their role in its ecological dissemination—there is limited evidence implicating birds directly.

**Response**: We are very grateful for your observation. We find interesting the possibility that birds are direct reservoirs of *H. capsulatum*. Thus, we have kept this idea since there are several studies where the presence of *H. capsulatum* is directly linked to birds such as: pigeons [1] and oilbird habitats [2] We have also included these references in the introduction section [3]

1.	Smith JL, Jones DB. Experimental Avian Ocular Histoplasmosis. Archives of Ophthalmology. 1962;67: 349–356. doi:10.1001/ARCHOPHT.1962.00960020351011

2.	Ajello L, Snow DW, Downs WG, Moore JC. Occurrence of Histoplasma Capsulatum on the Island of Trinidad, B. W. I.: I. Survey of Steatornis Caripensis (Oil Bird) Habitats. Am J Trop Med Hyg. 1962;11: 245–248. doi:10.4269/AJTMH.1962.11.245

3.	Teixeira M de M, Patané JSL, Taylor ML, Gómez BL, Theodoro RC, de Hoog S, et al. Worldwide Phylogenetic Distributions and Population Dynamics of the Genus Histoplasma. PLoS Negl Trop Dis. 2016;10: e0004732. doi:10.1371/JOURNAL.PNTD.0004732

Line 29 – “H. capsulatum presence is prevalent in wild animals, including rodents, marsupials, felines, xenarthrans, and others.” The statement is vague and lacks a clear take-home message. While it lists various wild animal groups associated with H. capsulatum, it doesn't explain the ecological, epidemiological, or transmission relevance of this information. Suggestion: “Histoplasma capsulatum has been found in wild mammals such as rodents, marsupials, felines and xenarthrans, suggesting diverse reservoirs that may influence its maintenance and transmission in endemic areas”. Please connect this to the next sentence.

**Response**: Many thanks to the reviewer. We have included your suggestion in the main text.

Line 34 – the term “protein gene” is not commonly used in formal scientific writing. “H. capsulatum was detected using a nested PCR assay targeting the 100-kDa protein-encoding gene”

**Response**: Thank you very much, we have included the change in the text.

Line 35 - Ecological niche modeling - Which Method?

**Response**: We have included the algorithm used for the ecological niche modelling experiment in this study in the abstract as requested: i.e., one class support vector machine (OC-SVM) hypervolumes. More details on this approach are available in the Methods section and the Supplementary Information 1. Thanks!

Line 40 – Improve conclusion. Suggestion: “This study documents the broad host range and ecological distribution of H. capsulatum in Ecuador, reinforcing concerns about its zoonotic potential. The detection of the fungus across diverse mammalian taxa and ecosystems emphasizes the importance of wildlife-based surveillance to better understand fungal pathogen reservoirs and geographic hotspots”

**Response**: Thank you very much for contributing to the article, we accept the suggestion and have included it in the main text.

Introduction
49 – Given the existence of multiple phylogenetic species within the Histoplasma genus, it is important to avoid generalizing H. capsulatum as a unique species.

**Response**: Many thanks to the reviewer, we agree with this comment. In fact, there are several species, and we have modified the introductory section accordingly (Lines 45 -48). "*Histoplasma* is a genus representing is a worldwide thermal dimorphic fungal pathogen mainly found across the Americas, Africa, and Asia [1]. One of the most important species of this genus, *H. capsulatum*, is considered a causative agent of histoplasmosis, which is acquired by inhalation of spores from contaminated soil”.

51 - The description of the infectious cycle omits a pivotal phase in the pathogenesis of Histoplasma spp.—the transition from the mycelial to the yeast form (dimorphism) upon entering the host. This thermally regulated shift is critical for intracellular survival and virulence, particularly within macrophages. I recommend revising the sentence to explicitly include this phase, as it represents a fundamental step in the fungal life cycle and host adaptation.

**Response**: Thank you very much for your comment, we have applied your suggestion in the text. Lines 48-51: “The infectious cycle involves the inhalation of infective mycelial spores which undergo a thermally regulated transition to the yeast form upon entering the host. This dimorphic shift is essential for intracellular survival and replication within macrophages and other phagocytes and is followed by the dissemination of the fungus back to the environment through carcasses, urine, and feces of infected animals”.

53 - the reintroduction of the fungus into the environment??

**Response**: We have improved the wording of this sentence to avoid misunderstandings. Please see the response above.

60 – The sentence referencing Brazil’s contributions to histoplasmosis research contains critical grammatical and structural issues. I recommend rephrasing it for clarity and accuracy.

**Response**: Thank you very much for your comment. We have modified and improved the sentence (lines 60-62): “Brazil has made significant contributions to histoplasmosis research, including reports of *H. capsulatum* isolation from various animals and soil samples across seven states”.

68 – The manuscript would benefit from a more comprehensive overview of the burden of histoplasmosis in Ecuador. As currently written, the mention of a single case gives the misleading impression that histoplasmosis is rare or poorly relevant in the country. However, this contradicts existing evidence. For instance, the study 10.1007/s10096-017-2928-5 highlights multiple cases of disseminated histoplasmosis in Ecuadorian patients with HIV, underscoring its clinical importance.

**Response**: We accept your suggestion. We have included your reference and other case studies. Now the paragraph has been improved as follows (lines 67-76): “In Ecuador, histoplasmosis has been historically underdiagnosed, although an increasing number of clinical reports suggest a broader distribution than previously recognized. In people living with HIV, a retrospective study documented multiple cases of disseminated histoplasmosis, highlighting its importance as an opportunistic infection [15]. Several hospital-based studies have further described probable and histologically confirmed cases in immunocompetent individuals presenting with respiratory or systemic symptoms in various regions of the country [16–18]. Additionally, a fatal pediatric histoplasmosis case was reported in 2023 involving prolonged fever, hemorrhagic diarrhea, and persistent anemia [19]. Moreover, a 2015 report described a dog presenting with multifocal lymphadenitis and mucocutaneous lesions, in which *H. capsulatum* was identified as the causative agent [20].”

Line 78 - The concept of Primary Pandemic Prevention (PPP), as described here, is important and timely. However, I suggest that framing this within a One Health context may be more appropriate and impactful. One Health emphasizes the interconnectedness of human, animal (both domestic and wild), and environmental health, and has been widely adopted as a guiding framework for pandemic preparedness and zoonotic disease surveillance.

**Response**: Many thanks to the reviewer and we agree, we have reworded the last paragraph of the introduction (lines 84-89): “The concept of Primary Pandemic Prevention (PPP), introduced in the aftermath of the COVID-19 pandemic, emphasizes the early detection and surveillance of pathogens in animal populations—particularly in wildlife—as a critical measure to prevent zoonotic spillovers. This perspective is inherently aligned with the One Health approach, which advocates for the integrated management of human, animal, and environmental health to address the complex dynamics of emerging infectious diseases [22]”.

Line 83 - The final paragraph of the introduction would benefit from a more impactful closing that clearly states the study’s main findings and the broader implications of the knowledge gap being addressed. I recommend adding a sentence that emphasizes both the novelty of detecting Histoplasma across a diverse range of wild mammalian hosts in Ecuador and the public health relevance of these findings.

**Response**: Thank you very much for your comment, we have improved the last paragraph of the introduction: “Within this framework, the zoonotic potential of *H. capsulatum* remains insufficiently characterized. The present study aims to contribute to this gap by identifying the presence of the fungus in tissues of wild mammals sampled across 19 provinces of Ecuador, spanning the Coast, Andean, and Amazon regions.”

Methodology
89 – 109

This section raises two important concerns that require clarification. Animal Handling and Necropsy Procedures: The manuscript does not provide sufficient detail on how animals were euthanized or how necropsies were performed. For studies involving the collection of tissue samples from wild mammals, it is essential to specify the methods used for euthanasia (if applicable), as well as the procedures and biosafety measures applied during necropsy. This is critical for reproducibility, ethical transparency, and to ensure compliance with international animal welfare standards. Legality of Armadillo Acquisition: The collection of armadillo specimens from hunters or rural markets raises potential ethical and legal concerns. In many countries, including Ecuador, hunting and trade of wild mammals—especially for bushmeat—may be prohibited or tightly regulated. The authors should clarify whether this practice was conducted legally and under appropriate authorization. If specimens were sourced without proper documentation, this could present a serious ethical issue.

**Response**: We appreciate and understand the reviewer's concern about the legal and ethical issues of wildlife collection. We have all the permits required for this study; therefore, we have detailed all the permits and protocols of each institution that collaborated in the research. Now in the paragraph of ethics and permits we have (lines 134-146): “All fieldwork and specimen collection were conducted in compliance with Ecuadorian environmental and bioethical regulations. The general collection was authorized under the framework contract for access to genetic resources, issued by the National Biodiversity Directorate of the Ministry of Environment, Water and Ecological Transition (MAATE). Bats were captured and euthanized following the protocols of the QCAZ Museum under permit MAAE-DBI-CM-2021-0165. Terrestrial rodents and other small non-volant mammals were collected and euthanized by INABIO, operating under permit MAAE-DBI-CM-2023-0334. In the case of armadillos, tissues were obtained from a previously authorized study (MAAE-DBI-CM-2021-0172) in which hunters donated armadillos’ organs. In Ecuador, indigenous communities in rural regions can legally use bushmeat as protein sources for subsistence, a common practice that it is recognized under national regulations.”

131 - a silica-based technique for nucleic acid purification?

**Response**: Yes, we used a silica-based technique for nucleic acid extraction and purification, using silicon dioxide (SiO_2_) powder in a guanidine–HCl solution, hereafter referred to as “Glassmilk.” We have improved this sentence to avoid any confusion: “The DNA extraction was conducted using glass milk methodology, a silica-based method which uses silicon dioxide (SiO_2_) powder in a guanidine–HCl solution [30].”

134 - What is Magic Blue???

**Response**: Magic Blue is a binding buffer composed by 20 mM Tris–HCl, 6 M Guanidine–HCl (MP Biomedicals) and 20% Triton X-100 (v/v), adjusted to a pH of 4.4. The solution is resuspend approximately 40 mg of silicon dioxide per milliliter of the binding buffer. We already modified this section with the denominations presented in the cited protocol (reference 30) to avoid misunderstanding.

134-136 – Could the authors clarify whether DNA precipitation and wash was carried out at room temperature or at a lower temperature (e.g., 4 °C or −20 °C), which is standard to enhance nucleic acid yield?

**Response**: Thanks for noticing this detail. DNA wash was carried out at room temperature, meanwhile DNA precipitation was performed at 4°C with an overnight incubation. We have amended this section.

137 - Could the authors specify the composition of the wash buffer used in the DNA extraction protocol?

**Response**: Thanks for noticing this detail. The composition of the wash buffer is presented in the cited protocol: Reference 30. In the same reference there is a step-by-step explanation of the extraction method and preparation of reagents used for this research project.

148 - Please rephrase the sentence describing the PCR setup for clarity.

**Response**: We have modified the methodology for clarity (lines 163-171): “For the identification of the 100-kDa protein-coding gene of *H. capsulatum*, a nested PCR was performed. For the first PCR, HcI (5-GCGTTCCGAGCCTTCCACCTCAAC-3) and HcII (5-ATGTCCCATCGGGCGCCGTGTAGT-3) primers were used to amplify a region of 391 base pairs (bp). For the second PCR, the primers used were HcIII (5-GAGATCTAGTCGCGGCCAGGTTCA-3) and HcIV (5-AGGAGAGAACTGTATCGGTGGCTTG-3), resulting in a PCR product of 210 bp. For this stage, the product of the first PCR was used as DNA template. Amplification was performed in reactions of 20 µL containing 1X GoTaq® Green Master Mix (Promega) and 0.4 µM of each primer. The PCR protocol was followed as stated by Bialek et. al [31].”

154 - The sentence refers to “BigDye 3.1® capillary electrophoresis matrix,” but this is technically inaccurate. BigDye Terminator v3.1® refers to the sequencing chemistry, not the electrophoresis matrix. Please revise the wording to accurately reflect that BigDye is the reagent used for Sanger sequencing, while the capillary electrophoresis matrix (e.g., POP-7 or POP-6) refers to the polymer used during fragment separation. Moreover, how PCR products were purified?? What is the size of the sequenced amplicons???? A lot of information is missing here.

**Response**: Thank you very much for your comment and we agree with what you mentioned. We have improved the text and included your suggestions (lines 174-177): “The second PCR products (210 bp) were sequenced using the Sanger technique on an ABI 3500xL Genetic Analyzer (Applied Biosystems, Foster City, CA, USA) with BigDye 3.1® Terminator and a capillary electrophoresis matrix. PCR products were purified by enzymatic purification with *Exo* I and FastAP [32].’’

Line 157 – It is unclear which sequences were used for comparison in the phylogenetic or identity analyses. I strongly recommend including a supplementary table listing all reference sequences used, along with their GenBank accession numbers, species identification, and relevant metadata.

**Response**: We agree with your comment. For this version we have removed the phylogenetic analysis considering that the Hcp100 sequences were phylogenetically uninformative as commented by the reviewer. For this version we have included Supplementary Table 3, which specifies the accession numbers, sample codes, and sequences of all *H. capsulatum*-positive samples. Thanks!

Line 177 - The manuscript mentions temperature, relative humidity, and chemical characteristics of soil as environmental predictors. However, it is not clear how and where these environmental data were obtained. Were they measured in situ at each sampling location, retrieved from public databases, or modeled?

**Response**: Thanks for this comment. As stated in the current version, details on each model parameter are available in the Supplementary material. All the environmental variables for the model were obtained from open repositories of climatic information, cropped, and further processed for the corresponding model. Specifically, we used MERRAclim, CHELSA, and SoilGrids repositories. Accordingly, we refer the reviewer to the supplementary material for further details of each repository, availability, and references for each data source. Thanks!

Results
Line 187 – indicate the total number of liver, spleen, lung, and feces samples

**Response**: Done, please see the results section.

Line 211 - The statement that this is the first documented evidence of Histoplasma capsulatum infection in two opossum, six rodent and 14 bat species species is inaccurate. Which species? Please name the hosts even mnentioning Table 1.

Response: We thank the reviewer for this observation. We agree that the original statement could be misleading without specifying the host species. We have clarified that, for Ecuador, this is the first report of H. capsulatum across such a wide range of hosts. To improve clarity, we have presented the scientific names of all positive host species in Table 1 together with an asterisk and footnote indicating those representing first records.

Line 221-230 –

While the use of short sequences in phylogenetic analyses is often challenging due to limited phylogenetic signal, it is not inherently problematic when handled with appropriate methodological rigor. In this context, I recommend that the authors explicitly state the total number of Hc100 sequences used for tree construction and discuss potential limitations associated with sequence length.
To independently evaluate the robustness of the presented phylogeny, I reconstructed a maximum likelihood tree using IQ-TREE2 with the command -m TEST and support values estimated by both aBayes and 1,000 ultrafast bootstrap replicates. The Ecuadorian strains appear to cluster into two clades; however, the branching pattern between these groups may be artefactual or reflect insufficient resolution.
The presented tree herein discussed shows two clades. Notably, the upper clade (labeled in blue), which contains the majority of the sequences, clusters more closely with the reference strain H67 (associated with the LAm A2 lineage; see Kasuga et al., 2003). In contrast, the lower clade (yellow) is more closely related to strain H66, which has been associated with the LAm B lineage (Kasuga et al., 2003). Still there is a bias on the sequence length here. I strongly encourage the authors to re-express the phylogenetic relationships in light of these findings and consult prior literature that more comprehensively characterizes these lineages—particularly the work [https://pubmed.ncbi.nlm.nih.gov/31372546/](https://nam10.safelinks.protection.outlook.com/?url=https%3A%2F%2Fpubmed.ncbi.nlm.nih.gov%2F31372546%2F&data=05%7C02%7Cfernanda.hernandez.alomia%40udla.edu.ec%7C42ced36a037c4f5d721208dd917560ff%7C585a4d92db1d4bbbb5acc5299e3894e3%7C0%7C0%7C638826657625920142%7CUnknown%7CTWFpbGZsb3d8eyJFbXB0eU1hcGkiOnRydWUsIlYiOiIwLjAuMDAwMCIsIlAiOiJXaW4zMiIsIkFOIjoiTWFpbCIsIldUIjoyfQ%3D%3D%7C60000%7C%7C%7C&sdata=3VbQY52ws2xlLOGFHIKZXmPqS6lr%2BlU88sdM9spz%2FF4%3D&reserved=0), which provides deeper insights into the genetic structure and population differentiation of colombian strains and is comparable to your dataset. See attached files

Another possibility is mention that strains are grouped into two different phylogenetic clusters without mentioning their genetic background.
I have no concerns about the species niche modeling.

Response: Thanks for your careful assessment of our phylogenetic analysis. We analysed your results in detail and we can appreciate the relevance of your suggestions. However, for this version, we decided to eliminate our phylogenetic approximation following this reasoning: as our sampling is inherently biased since the collection was done for biodiversity assessments of the species and a systematic effort for histoplasmosis in wildlife was lacking, the few sequences recovered might offer only a partial picture in terms of *H. capsulatum* phylogeny. Moreover, reviewing the literature we can see that our sequence lacks enough phylogenetic signal to reconstruct robust trees for interpretation. Thus, we have limited our study to describe the presence of *H. capsulatum* across different mammals’ orders which we can defend robustly and that it is the true goal of our research. Thanks!

Discussion

The discussion is generally well-written and informative; however, it would benefit from a deeper contextualization of the findings within a One Health framework. In particular, I recommend expanding the section to include more information on the burden and epidemiology of histoplasmosis in humans in Ecuador and neighboring regions. There is ample evidence that histoplasmosis is endemic in Ecuador, especially in immunocompromised individuals, including people living with HIV. Integrating this information would help situate the findings on opossum infection within a broader ecological and public health context, supporting the relevance of the study to One Health initiatives and pathogen surveillance strategies in the region.

**Response**: Thank you very much for the comment. We have included a paragraph in reference to One Health perspectives. Here, we offer a deeper contextualization of the study implications in both public health and conservation.

**Reviewer #2:** While I think this could be a great contribution to the literature on Histoplasma in Ecuador, it needs significant improvement. In the introduction, there is a statement that birds and bats transmit Histoplasma via fecal route. While it is true that guano piles in caves and coops have been implicated sources of infection during outbreaks, there is still much debate on carriage. For the few studies that have directly collected from living animals, the rate of positivity is still low or zero. Dead animals are also found in guano piles thus Histoplasma could grow out of infected tissue, rather than dispersed via fecal route.

**Response**: We are very grateful for your important comment. We agree that the statement in the introduction, where it is suggested that birds and bats can transmit *H. capsulatum* by fecal route, could lend itself to interpretations that exceed the direct evidence available, especially considering that the studies that have evaluated the active excretion of the fungus in live animals are scarce and, in many cases, have reported low or zero positivity rates. However, we are interested in the possibility that certain birds may act as direct reservoirs of *H. capsulatum*. In this context, we would like to maintain this idea in the manuscript, supported by previous studies that have linked the presence of the fungus to environments directly associated with birds, such as pigeons (*Columba livia*) [1], guacharo or oilbird (*Steatornis caripensis*) habitats [2]. In addition, we have included a recent review compiling evidence on this association [3], which has been incorporated in the introduction section to strengthen the bibliographic support.

Finally, in response to your comment, we revised the wording of the corresponding section in the introduction to reflect, in a more balanced way, the current state of knowledge on this topic, avoiding categorical statements and highlighting the different hypotheses under discussion.

1. Smith JL, Jones DB. Experimental Avian Ocular Histoplasmosis. Archives of Ophthalmology. 1962;67: 349–356. doi:10.1001/ARCHOPHT.1962.00960020351011
2. Ajello L, Snow DW, Downs WG, Moore JC. Occurrence of Histoplasma Capsulatum on the Island of Trinidad, B. W. I.: I. Survey of Steatornis Caripensis (Oil Bird) Habitats. Am J Trop Med Hyg. 1962;11: 245–248. doi:10.4269/AJTMH.1962.11.245
3. Teixeira M de M, Patané JSL, Taylor ML, Gómez BL, Theodoro RC, de Hoog S, et al. Worldwide Phylogenetic Distributions and Population Dynamics of the Genus Histoplasma. PLoS Negl Trop Dis. 2016;10: e0004732. doi:10.1371/JOURNAL.PNTD.0004732

**Reviewer #3:** In regarding to the revision of the Manuscript MS PNTD-D-25-0030, entitled
"Histoplasma capsulatum in Wild Mammals from Ecuador", I would like to
emphasize its importance in the epidemiology of histoplasmosis, mainly
considering a wide sort of animals studied (106 species) and the role of wild
mammals as possible reservoirs and, particularly, the role of bats as the main
reservoir and disperser of the fungal pathogen H. capsulatum in nature, as have
been suggested by some researchers. Thus, any type of evidence for the
occurrence of H. capsulatum in wild mammals, either in Latin America or in other
regions or the world should be privileged. However, I would like to question the
efficacy of the Hcp100 molecular marker for the phylogenetic analysis. The Hcp100
molecular marker is successfully used in clinical diagnosis and for searching the H.
capsulatum presence in randomly infected animals, because this marker amplified
a specific DNA fragment of 210 bp, in the second Nested-PCR reaction, which is
unique for this fungal pathogen. Although its use for H. capsulatum phylogenetic
analyses is not the most recommendable, mainly considering that the Hcp100
fragment presents very low polymorphism, clades obtained were certainly
representative and discriminate the different groups of animals studied. Hence, I
suggest including appropriate interpretations in the Discussion section of the
Manuscript.
Overall, considering the above-comment, I think that the Manuscript must be
accepted.

**Response**: This comment was previously responded in the methods section commentaries.
